# Body Image and Body Experience Disturbances in Schizophrenia: an Attempt to Introduce the Concept of Body Self as a Conceptual Framework

**DOI:** 10.1007/s12144-016-9526-z

**Published:** 2016-11-25

**Authors:** Olga Sakson-Obada, Paulina Chudzikiewicz, Daniel Pankowski, Marek Jarema

**Affiliations:** 1 Institute of Psychology, Adam Mickiewicz University, 89 Szamarzewskiego Street, 60-568 Poznan, PL Poland; 2 III Psychiatric Clinic, Institute of Psychiatry and Neurology, Sobieskiego 9 Street, 02-957 Warsaw, PL Poland; 3 Faculty of Psychology, University of Warsaw, Stawki 5/7 Street, 00-183 Warsaw, PL Poland

**Keywords:** Body self, Body image, Psychosis, Schizophrenia

## Abstract

Disturbances in body experience are described as key schizophrenia symptoms and early disease predictors. In case studies, different disorders relating to body experience are presented, but only a few empirical studies have aimed to distinguish the characteristics of body experience in schizophrenia, and these have been selected arbitrarily and without reference to cohesive theoretical model. To integrate this fragmentary approach, we propose a body self (BS) model, composed of: functions; representations (e.g., body image); and sense of body identity. The aim of the study was to determine whether the BS differentiates schizophrenic patients from healthy controls, and to investigate the relations between aspects of BS and a history of illness and clinical characteristics. The Body Self Questionnaire and the Positive and Negative Syndrome Scale were administered to 63 schizophrenic patients and 63 healthy subjects. The difference was found in the functions of the body-self (perceiving, interpreting, and regulating body experience), in the sense of body identity, and in one of three aspects of body image explored (e.g., acceptance of biological sex). Disturbances in BS were related to positive symptoms and to the number of hospitalizations for other diseases. Together, the results demonstrate that schizophrenia is more body experience than body image disorder, since the negative emotional attitude towards the body and acceptance of fitness were not distinctive for schizophrenia. The link between the disturbances in BS and the number of nonpsychiatric hospitalizations suggests that misinterpretation of body experiences in schizophrenia can promote a search for medical attention.

## Introduction

The classic publications of Bleuler (1950) and Kraepelin (1916) describe numerous disorders relating to body experience as key symptoms of schizophrenia. Such symptoms used to be categorized as early disease predictors experienced by 50 % to 70 % (Ferri et al. 2014; Stanghellini et al. 2012). Disturbances in body perception, such as seeing another person or a distorted face in one’s reflections (Rosenzweig and Shakow 1937; Harrington et al. 1989), acute changes in sensing, where normal colors and sounds become painful while tastes and smells are perceived in a dulled manner (as Luis (1974) put it: “everything tastes like sawdust”), rejection of an aspect of the body, expressed in major self-mutilation without the expression of pain (Large et al. 2009); and abnormal cenesthopathic sensations (Jenkins and Rochricht 2007; Murawiec 2013) are just a few examples of the disorders of body experience in schizophrenia that have been described by clinicians or patients themselves. Disturbances of body experience in schizophrenia thus constitute a wide range of phenomena for which no common theoretical denominator has yet been found. This state of knowledge is also reflected in the fragmented empirical studies (e.g., studies that take into account a single or a few arbitrarily selected aspects) using different assessment methods (self-report measures, experiments, etc.).

### Body Image and Body Experience in Schizophrenia - Results of Empirical Studies

Unlike case studies, some empirical studies have aimed to distinguish general characteristics of body experience in schizophrenia that could be considered specific to this disorder. Generally speaking, such studies cover three fields of research: distortion in body experience, attitude to one’s appearance, and stimulus processing. The first two are based on the body image concept, discussed widely in the literature (Schilder 1935; Cash 2011). Unfortunately, researchers do not define the term, which results in aspects being chosen somewhat haphazardly for examination. For example, Koide et al. (2003) distinguish through factor analysis three components of body image: anatomical (relating to body shape), functional (e.g., powerlessness or strong gastrointestinal function), and psychological (satisfaction with appearance, lifelessness) and Chapman et al. (1978) list different body image aberrations, varying from the sense of losing body boundaries to size changes in the body or its parts. Generally, considering the results of such studies in the context of distorted body experience (e.g., boundary loss, depersonalization), Chapman et al. (1978) and Koide et al. (2003) both using the self-report method, demonstrated differences in the aspects studied between schizophrenia subjects and healthy people, whereas Priebe and Rohricht (2001) generally did not notice such differences when comparing with a group of patients hospitalized for other disorders. Caputo et al. (2012), using experimental procedures, found that schizophrenic patients more often than controls experience depersonalization in terms of distortion of their faces after gazing at their reflection for some time. Moreover Stanghellini et al. (2014) introducing the concept of bodily self rooted in a phenomenological approach, distinguished unique pattern of body phenomena in persons with schizophrenia. The core categories of distorted body experience in this study were: dynamization (disturbances of bodily boundary), morbid objectification (body experienced as devoid of life), and dysmorphic or pain - like phenomena.

The second aspect of body image studied is emotional attitude towards appearance. In their research based on the self-report method Koide et al. (2003) found no differences between schizophrenics and controls, whereas Priebe and Rohricht (2001) showed that schizophrenics were more satisfied with their appearance than patients with anxiety and depressive disorders.

In turn, the empirical verification of clinical reports describing the radical disturbances in stimulus perception observed in schizophrenia was based on experimental methodology. Studies have shown the following changes in sensations processing in schizophrenics, compared to controls: reduced ability to distinguish odors (Cumming et al. 2011) and their evaluation in terms of edibility (Rupp et al. 2005), and reduced sensitivity to proprioceptive stimuli (Rosenbaum et al. 1965) and pain (Blumenshon et al. 2002; Boettger et al. 2013). There are, however, studies that failed to confirm differences in pain thresholds between healthy people and schizophrenics (Bonnot et al. 2009). Based on these results, it may be hypothesized that schizophrenia generally leads to elevated sensory thresholds; however, various sense modalities have not yet been considered in a single study. Moreover Priebe and Rohricht (2001) measured body size perception due to tactile stimuli and proved, that schizophrenic patients underestimated the size of legs. Although it is not clear why the disturbances occurs only when the size of legs was estimated, the obtained results were explained in the light of dysfunction of perception (e.g., sensory information processing).

Unfortunately, empirical studies aiming to identify specific features of schizophrenic disorders in body experience tend to choose several aspects without reference to any cohesive theoretical model. To integrate this fragmentary approach to body experience in schizophrenia (and in psychopathology research in general), we propose the body self model (Sakson-Obada 2009; Sakson-Obada and Wycisk 2015; Kubiak and Sakson-Obada 2016).

### The Body Self Model - an Attempt to Build a Bridge between Different Conceptual Approaches to the Body-Mind Issue

The body self a concept developed by one of the authors (2009, 2013), unites two different approaches to body-mind phenomena. The first underlines the body as the object of perception and affective evaluation, referred to as body image (e.g., Schilder 1935; Cash 2011). The second approach, based on the assumption that body experience is the core dimension of identity (Stanghellini et al. 2014; Krueger 2002; Allport 1960) or cognition (e.g., Barsalou et al. 2003), emphasizes the position of experiencing subject. The body self model thus covers both the body image and body experience aspects and builds a bridge between the two theoretical lines of the „body-mind issue” - namely the body as an object (body image), and the body as a core aspect of self-experience.

**Fig. 1:**
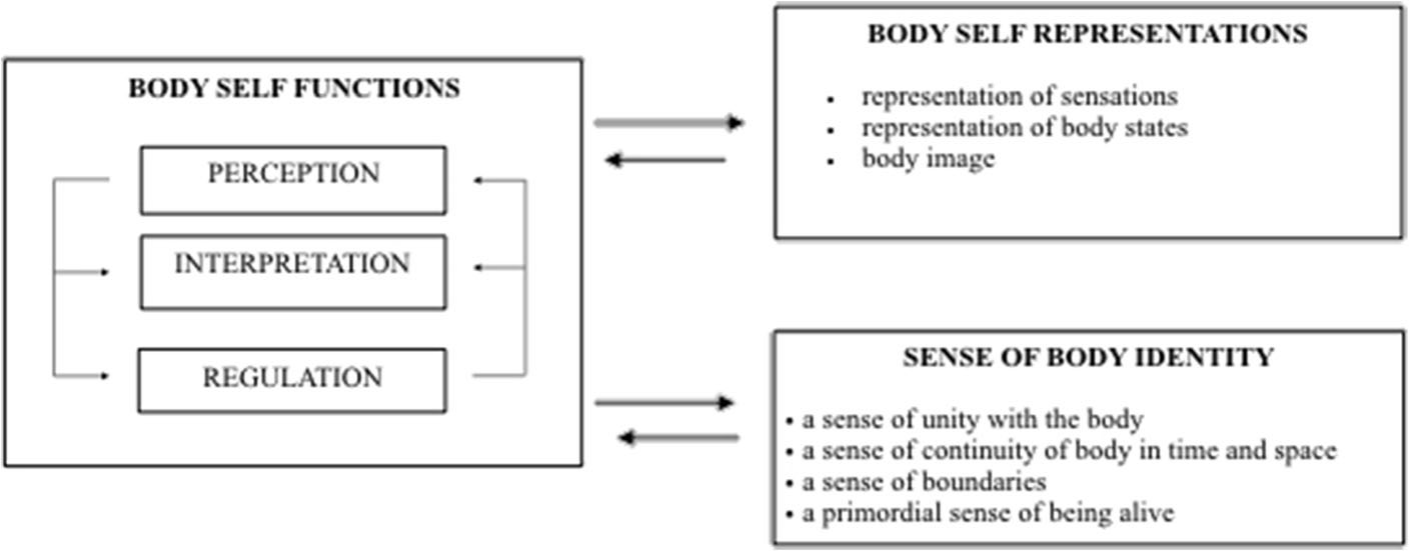
Body self model

The body self (BS) is a complex, tridimensional aspect of personality composed of: (1) functions, (2) the sense of body identity, and (3) representations of sensations, body states, and body characteristics (e.g., body image) [Fig. 1]. It was assumed that the body self is the aspect of personality that organizes body experiences in the form of representations. The content and the formal aspects of representations (e.g., the level of organization, differentiation, and stability) depend on particular functions of the body self, namely: perception of stimuli and their integration in the form of sensorimotor representations, organized in a new order, when the process of verbalization is operative**.** In other words, when a child enters the world of words, he or she can interpret experience using language, thus promoting adequate interpretation of internal states and more mature strategies of coping with them (cf. the process of desomatization and symbolization of affect introduced by Krystal (1979) and Krueger (2002)). It is also assumed that consolidation of body experience is based on the caregiver’s ability to offer adequate interpretation of the child’s experience not only in the form of reaction (such as comforting when the child is anxious) but also in process of proper naming the internal states of the child (Stern 1985). Moreover, the naming of the child’s body characteristics by the caregivers make the visual representation of the appearance more precise and differentiated. Consequently, three basic functions of body self are distinguished: 1) perception of stimuli coming from interoceptors (e.g., heartbeat) and exteroceptors (e.g., touch, odour), 2) interpretation of body experience in terms of emotions or physical needs, and 3) regulation of emotions and physical needs.

It is also assumed that the ability to feel, understand, and cope with bodily experiences in a sufficient and correct manner should be reflected in both the adequate representation of the body and its characteristics and the sense of body identity. In a case of a healthy, adult person such aspects of sense of bodily identity as (a) unity with the body, (b) continuity of the body in time and space, (c) boundaries of the body, and (d) the primordial sense of being alive, are not consciously perceived in the categories of any aspect of the *self*. A number of authors have assumed that a normal relationship between body and mind entails the state of quiet cooperation’, where the bodily functioning does not preoccupy the attention of an individual (Erikson 1956; Stanghellini et al. 2012).

The final dimension of the body self discussed refers to representations of sensations, body states and body characteristics. Thus the concept of body image, widely discussed in the literature (Schilder 1935; Cash 2011), refers to affective and cognitive representations of different body characteristics mainly related to the appearance. Development of the body image as an affect-laden representation depends on caregivers’ attitudes toward the child’s body, which is expressed not only in words, but also in nonverbal communication (such as in the way the caregiver touches and looks at a child). Social comparison with peers also play a role in the development of body image during adolescence, when the body image is a pressing issue (Tatangelo and Ricciardelli 2015). Further some scholars claim that the ideal of thinness promoted by the media may also influence body image. However, the results of the latest studies weakened the thesis about direct media influence on body dissatisfaction, proving that the peer competition is a key risk factor for body dissatisfaction (Ferguson et al. 2014).

Three aspects of the body image are included in the model: satisfaction with appearance, satisfaction with fitness, and acceptance of biological sex; however the spectrum of body representations potentially considered is very broad.

In sum, the body self is a complex three-dimensional structure of personality that introduces bodily experiences on the mental plane. The BS model has been positively verified in a few empirical studies. Studies conducted in a healthy population, trauma survivors, and subject with insecure attachment showed strong relationships between all functions of BS, functions and both the sense of body identity and satisfaction with appearance, and moderate relationships between representations of BS (Sakson-Obada 2009). The Body Self Questionnaire, based on the model of BS, succeeded in differentiating a normal population and a clinical groups in which disturbances in the body self were assumed (Pyrgiel 2014; Kubiak and Sakson-Obada 2016).

### Summary and Aim

The body self model allows integration of observations from the field of body experience and body image in schizophrenia. Taking the body self conceptualization as starting point, it seems justified to conclude that results of the studies relate to the sense of body identity (e.g lifelessness, body boundary, depersonalization), to one type of body image representation (satisfaction with appearance), and to the function of perception of stimuli (e.g. pain, olfactory perception; see: Table 1). The aim of the study was to determine if the three-dimensional body self model differentiates schizophrenic patients from healthy controls, and to investigate relations between aspects of body self and a history of illness and clinical characteristics (positive and negative symptoms, general psychopathology).

**Table 1:** Disturbances in the body self in schizophrenia

Aspect of body self	The aspects examined
Functions	
Perception	
• Qualitative disturbances	Pain - like phenomena (Stanghellini et al. 2014), cenesthopathic sensations (Jenkins and Rochricht 2007; Murawiec 2013)
• Quantitative disturbances	Reduced sensitivity to proprioceptive stimuli (Rosenbaum et al. 1965) and pain (Blumenshon et al. 2002; Boettger et al. 2013), reduced ability to distinguish odors (Cumming et al. 2011)
Sense of body identity	
A sense of having boundaries	A sense of boundaries (Chapman et al. 1978; Priebe and Rohricht 2001), dynamization (Stanghellini et al. 2014)
A sense of continuity in space	Changes in body size (Chapman et al. 1978; Priebe and Rohricht 2001)
A sense of continuity in time	Changes in body appearance (Chapman et al. 1978)
A sense of unity with the body	Depersonalization, a sense of body strangeness (Chapman et al. 1978; Caputo et al. 2012)
A sense of being alive	A lack of vitality (Koide et al. 2003), morbid objectification (Stanghellini et al. 2014)
Body self representation	
Body image	
• Cognitive aspect	Beliefs regarding the body size, appearance (Koide et al. 2003; Priebe and Rohricht 2001)
• Affective aspect	Dissatisfaction with appearance (Koide et al. 2003; Priebe and Rohricht 2001)

## Participants and Methods

### Participants

Sixty-three participants with diagnoses of schizophrenia took part in the study (58.7 % male and 41.3 % female; age M = 29.5; SD = 5.5), after obtaining written informed consent to the protocol from the local ethics committee. The remaining demographic and clinical characteristics are shown in Table 2. Diagnosis by ICD-10 criteria was based on medical records; no additional verification was made. Patients were recruited from a psychiatric rehabilitation ward F-9, Clinic of Psychiatry and Neurology Institute, Warsaw. This ward provides psychiatric treatment and rehabilitation for 18–35 year-old patients with diagnosis of schizophrenia and other psychotic disorders. The control group without mental disorders and suicide ideation was sex- and age-matched. Exclusion criteria for the participation in the study were: intellectual disabilities and presence of disease that could affect body experience.

**Table 2:** Demographic and clinical characteristics of subjects

Demographic characteristics			
	Schizophrenia (*n* = 63)		Control group (*n* = 63)
Relationship status (n, %)			
Single	52 (83 %)		20 (32 %)
Partnership	11 (17 %)		43 (68 %)
Sex			
Male	37 (58.7 %)		37 (58.7 %)
Female	26 (41.3 %)		26 (41.3 %)
Age (SD)	29.5 (5.5)		28.3 (5.4)
Mean years of education (SD)	16.8 (2.3)		18.5 (3.7)
Body Mass Index (BMI)	26.8 (6.7)		23.2 (3.1)
Clinical characteristics			
	Mean	SD	
Age of onset	21.2	5.7	
Relapse number	3.3	3.6	
Duration of illness (years)	7.8	6.7	
Time since last episode (months)	10	25.3	
Number of hospitalization due to schizophrenia (in last 12 months)	1.8	0.9	
Number of hospitalization due to other diseases (in last 12 months)	0.2	0.6	
PANSS			
Positive scale	9.8	0.5	
Negative scale	10.9	5.8	
General psychopathology	23.2	7.1	

### Methods

Sociodemographic data (gender, age, years of education, body–mass index (BMI), current diseases, relationship status), the patient’s disease history, and the subjective side effects of antipsychotic treatment were obtained through the questionnaire completed by the subjects.

The Body Self Questionnaire (BSQ) assesses the aspects of BS: namely, the functions, body image, and the sense of body identity (Sakson-Obada 2009; Saks on-Obada and Wycisk 2015). It is made up of 78 (81) statements in the version for women (men) and assesses disturbances in BS functions (perception, interpretation, regulation), sense of physical identity, and three aspects of body image (appearance evaluation, fitness evaluation, acceptance of biological sex). There are two subscales measuring perception disturbances—Lowered threshold (7 items, e.g., Sometimes even a gentle touch is painful) and Heightened Threshold (10 items, e.g., Sometimes all or part of my body is insensitive to pain), one measuring the ability to interpret emotions and bodily needs (13 items, e.g., It’s difficult for me to find the right words for my feelings), and one assessing the regulation of emotions and physiological needs (20 items, e.g., Sometimes I cannot stop eating, even though I am not hungry). The Sense of body identity scale contains 10 items (e.g., Sometimes I feel dead inside). These scales are the same in both the male and female versions. Three subscales evaluate the body image dimension: Appearance (8 items, e.g., I feel physically attractive); Fitness (5 items for women, 6 for men, e.g., I have good motor coordination); Acceptance of biological sex (5 items for women, 7 for men, e.g., I’d rather be a person devoid of sex attributes). Answers are given on a 5-point Likert scale (1: not true at all; 5: very true). Higher scores reflect a greater number of disturbances in the BS.

PANSS (Positive and Negative Syndrome Scale, Kay et al. 1987) is a 30-item clinical tool assessing schizophrenia symptoms. Each item is rated from 1 (absent) to 7 (extreme). The scale contains three subscales: positive symptoms (7 items), negative symptoms (7 items), and general psychopathology (16 items).

Differences between the control and experimental groups were computed using one-way ANCOVA, controlling for the selected socio-demographic variables. Effect sizes (Cohen’ d) were also reported. Further, correlations between PANSS and BSQ were obtained using Pearson’s *r* coefficient. Stepwise multiply regression analysis were performed in an attempt to indicate predictors of disorders in the body self in schizophrenia group. Assumed statistical significance was *p <* 0.05, two-tailed. Data analysis employed PS IMAGO SPSS 22.

## Results

### Analysis of Differences in the Body Self in Schizophrenia and Control Group

The basic statistical description of demographic and medical variables is presented in Table 2. The frequencies in Table 2 indicate that schizophrenic patients are more often single than healthy controls (χ^2^ = 35.14, df = 1, *p* < .001), have fewer years of education (*t* = −4.36, df = 124, *p* < .001), and have higher BMIs (*t* = 3.85, df = 124, *p* < .001) than the control group. All patients were undergoing antipsychotic medication (51 patients on first-generation antipsychotics (FGAs), 5 patients on both first-generation and second-generation antipsychotics (SGAs), and 7 patients on a drug adjustment procedure). Considering the potential detrimental effect of medication on sensitivity, weight gain, emotion, and physical states (e.g., sedation, increased appetite, and somnolence), the subjective side effects of medication were controlled for. More than half the patients (58.7 %) reported side effects of antipsychotic medication, and 22 % reported more than one symptom. The most frequented side effects mentioned were: somnolence (6 subjects), weight gain (5 subjects), physical weakness or sedation (5 subjects), and muscle stiffness (4 subjects). Small numbers of patients mentioned other side effects: agitation (2 subjects), headache (2 subjects), agitation (2 subjects), and sexual dysfunction (2 subjects). To assess the potential effect of medication on the body-self, we compared patients who had mentioned side-effects of medication with those who had not. This comparison revealed no statistically significant differences between the two groups in any of the BS variables investigated, so in further analysis, the side effect variable was not included.

Analysis of covariance (ANCOVA) was used to compare the difference between schizophrenic patients and healthy controls in the BSQ results, controlling for relationship status, years of education, and BMI. The analysis showed that the results for each BSQ subscale measuring functions of the body self and the sense of body identity were significantly higher in schizophrenic patients (see Table 3) who thus, more often than controls, experienced both increased and decreased sensitivity to external stimuli; however the difference is smaller for lowered thresholds. Moreover, schizophrenic patients had more difficulties interpreting and coping with emotions and bodily states (e.g., hunger and sexual arousal) than the control group. They also struggled with body identity disorders—like the loss of body boundaries or failure to recognize themselves in the mirror—more often than the control group. Only one aspect of body image—the acceptance of biological sex—differentiated the two groups. Schizophrenic patients declared more negative emotions (such as shame and disgust) toward the sexual attributes of their body than did healthy controls. The groups did not significantly differ in terms of satisfaction with appearance and fitness.

**Table 3:** Differences between groups in BSQ scores using ANCOVA with years of Education, BMI, and relationship status, as covariates

BSQ scales	Schizophrenia		Control group		F	df_2_	p	Cohen’s d
	M	SD	M	SD				
Heightened threshold	1.68	0.59	1.39	0.40	7.54	121	.007	.57
Lowered threshold	1.79	0.66	1.54	0.49	6.42	121	.013	.42
Interpretation	2.54	0.77	2.04	0.52	8.25	121	.005	.76
Regulation	2.81	0.69	2.29	0.54	8.61	121	.004	.83
Sense of body identity	1.95	0.78	1.37	0.50	5.07	121	.026	.87
Satisfaction with appearance	2.26	0.91	1.94	0.62	2.45	121	.12	.41
Satisfaction with fitness	2.66	1.00	2.27	0.65	1.52	121	.193	.47
Acceptance of biological sex	1.50	0.56	1.23	0.37	5.41	121	.021	.57

A large effect in BSQ regulation and sense of body identity was observed, whereas a medium-sized effect was detected for heightened threshold, interpretation, and acceptance of biological sex. Lowered threshold, evaluation of appearance, and evaluation of fitness, as measured by BSQ, had small effect sizes.

### Analysis of Body Self Correlates in Schizophrenic Group

Table 4 presents Pearson’s *r* correlation coefficient between the PANSS and BSQ subscales. No BSQ subscales were significantly correlated with the PANSS negative symptoms subscale. Associations between positive symptoms and a few aspects of BS were observed. Positive symptoms (delusions, excitement) were related to heightened thresholds, difficulties in regulation of emotions and bodily states, disorder in the sense of body identity, and dissatisfaction with appearance. Only one aspect of the BS sense of body identity was positively correlated to general psychopathology, as measured by PANSS.

**Table 4:** Correlations between PANSS and BSQ subscales

	PANSS Positive Symptoms	PANSS Negative Symptoms	PANSS General Psychopathology
Heightened Threshold	.36**	-.03	.14
Lowered threshold	.19	-.06	.06
Interpretation	.04	-.01	.17
Regulation	.27*	.06	.21
Sense of body identity	.35**	.20	.44***
Satisfaction with appearance	.28*	.11	.24
Satisfaction with fitness	.20	.11	.20
Acceptance of biological sex	.16	.20	.23

*-*p* < 0,05;**-*p* < 0,01;***-*p* < 0,001; BSQ: The Body–Self Questionnaire; PANSS: Positive and Negative Syndrome Scale

Table 5 shows the correlations between BSQ subscales and demographic data (BMI, age, years of education, relationship status, and sex) and course-of-schizophrenia variables (age of onset, duration of illness, number of relapses, time since last episode, number of hospitalizations for schizophrenia, number of hospitalizations for other diseases). A negative correlation was observed between age and heightened and lowered threshold. Years of education were related to heightened and lowered threshold, interpretation, regulation, and sense of body identity, whereas age of onset correlated negatively only with the sense of body identity. No difference emerged between single and partnered individuals patients in regard to the BS variables. Moreover, women, as compared to men, received higher scores in regulation (*M* female =3.1, *M male* = 2.6*; t* = 2.6, *p* = .01), satisfaction with appearance (*M* female =2.8, *M* male =1.9*; t* = 4.3, *p* = .001), and satisfaction with fitness (*M* female =3.0, *M* male =2.4*; t* = 2.3, *p* = .03). Hospitalization for other diseases was related to disturbances in perception (heightened and lowered threshold) and regulation.

**Table 5:** Correlations between BSQ subscales and demographic and course-of-schizophrenia variables

	BMI	Age	Years of education	Age of onset	Duration of illness	Relapse number	Time since last episode	Number of hospitalizations due to sch.	Number of non- psychiatric hospitalizations
Heightened threshold	-.50	-.20*	-.23**	-.03	-.15	-.07	-.23	.05	.35***
Lowered threshold	.30	-.18*	-.19*	-.08	-.04	.09	-.26	-.07	.27**
Interpretation	0.06	-.15	-.19*	-.10	.07	.24	-.17	.03	.11
Regulation	.14	-.04	-.23*	-.15	.25	.22	-.12	.01	.20*
Sense of body identity	.12	-.12	-.32***	-.30*	.21	.13	-.18	.13	.15
Satisfaction with appearance	.07	-.01	-.08	-.01	.05	-.06	-.23	-.02	.25
Satisfaction with fitness	.17	.11	-.10	.01	.15	.05	-.30	.24	.12
Acceptance of biological sex	.02	-.03	-.09	-.10	-.05	.13	-.13	.10	.07

*-*p* < 0,05; **-*p* < 0,01;***-*p* < 0,001; BSQ: The Body Self Questionnaire;

These results were verified using stepwise regression analysis. Nonpsychiatric hospitalization was the only predictor for two aspects of body-self: lowered threshold (*R* ^2^_adj._ = 0.15; *F* (1,24) = 5.46; *p* = 0.03; *β* = 0.37, SE = 0.16; *p* = 0.03) and heightened threshold (*R* ^2^_adj._ = 0.33; *F* (1, 24) = 13.29; *p* = 0.001; *β* = 0.48, SE = 0.13; *p* = 0.001). Gender was the only predictor for the interpretation (*R* ^2^_adj._ = 0.13; *F* (1,24) = 4.67; *p* = 0.04; *β* = −0.66, SE = 0.30; *p* = 0.04), which means that female patients described more problems with the interpretation of physical states and emotions than did male patients. Gender and age of onset were the best predictors for regulation (*R* ^2^_adj._ = 0.42; *F* (2, 23) = 10.03; *p* = 0.001; *β*_*gender*_ = −0.85, SE = 0.23; *p* = 0.001; *β*_*onset*_ = −0.64, SE = 0.02; *p* = 0.01). The results revealed that both female gender and early onset of schizophrenia are risk factors for difficulties with emotional regulation and physical needs. Gender and positive symptoms were the best predictors (female gender being a risk factor) for dissatisfaction with appearance *R* ^2^_adj._ = 0.48; *F* (2, 23) =12.48; *p* = 0.001; *β*_*gender*_ = −1.44, SE = 0.39; *p* = 0.001; *β*_*positive*_ = −0.92, SE = 0.38; *p* = 0.024). The analysis did not distinguish any significant predictors for sense of body identity, fitness evaluation, or acceptance of biological sex. Stronger correlations between the predictors than between the predictors and the dependent variables may explain the fact that, in the regression analysis (i.e., the method with more rigorous requirements), fewer results turned out to be significant than in the correlation analysis. For this reason, the discussion was based mainly on the results of the correlation analysis, in order to capture more detailed relationships between the variables.

## Discussion

The first part of the study attempted to determine differences between schizophrenic patients and healthy controls in BS, considered as a complex, three-dimensional aspect of personality. The results revealed significant differences in each BS function and sense of body identity, whereas differences in representations (body image) were insignificant, with the exception of acceptance of biological sex.The basic function of the BS is the perception of stimuli, and schizophrenic patients indicated that they suffered from more disturbances than controls in terms of both lowered and heightened thresholds of sensation. Schizophrenics’ insensitivity to sensations - including pain, proprioceptive stimuli, and odors - were confirmed in experimental research (Blumenshon et al. 2002; Boettger et al. 2013) and is supported by our results. One intriguing result refers to the lowered threshold of sensations in schizophrenic patients, compared to controls. However, the idea of hypersensitivity was recently developed by a few authors and has been described as a sensorimotor gating disturbance in schizophrenia (Keil et al. 2016; Smucny et al. 2013). This cognitive disorder is based on a deficit in preconscious information processing, considered as an attentional abnormality leading to stimulus overload and cognitive fragmentation, expressed phenomenologically as “being attacked by sensations”. This hypothesis was verified by Smucny et al. (2013), who demonstrated that schizophrenic patients are easily distracted by normal sounds and have difficulties concentrating on tasks; our study confirmed this, revealing that patients can register and express this problem through the declarative method. As a lowered and a heightened threshold are strongly interrelated in the schizophrenic group (*r* = 0.714; *p <* .001), we can conclude that schizophrenic patients experience alternating states: being sometimes oversensitive to stimuli (perceiving them as painful or irritating) and sometimes perceiving them as dulled.

Schizophrenic patients also experience more difficulties interpreting and coping with emotions and physical needs. They thus have problems identifying bodily states correctly and indicating the cause of emotional and physical states (like hunger, illness) and coping with them. The results agree with those of Henry et al. (2010), who demonstrated higher levels of alexithymia in a schizophrenic group than in controls. The confusion in sensation, emotions, and bodily states was reflected in disorders in the sense of body-identity diffusion (all of function variables were related to the sense of body identity variable; *r* values ranged from *r* = .43, *p* < .01 to *r* = .65, *p* < .01). Schizophrenic patients declared more disturbances in this aspect, which included e.g., a sense of internal death, dissolution of body boundaries, and not recognizing one’s reflection in the mirror.

Moreover, only one aspect of body image differentiate the clinical and control groups. That fact the greatest difference was obtained in the acceptance of biological sex suggests that schizophrenic patients suffer from problems incorporating the body attributes that define them as male or female into their self-concepts (c.f. Nasser et al. 2002; Rajkumar 2014). Interestingly, although the average BMI was higher in the schizophrenics than in the controls, the evaluation of fitness did not differentiate the two groups. Our results do not agree with those obtained by Vancampfort et al. (2011), who found such a difference when comparing schizophrenic and control groups. According to Vancampfort et al. (2010, 2011), decreased physical self-perception in schizophrenia patients, together with their lower physical fitness parameters, are related to different factors, such as negative symptoms linked with a sedentary lifestyle, low self-efficacy, sleep problems, and low physical self-worth (Vancampfort et al. 2010; Vancampfort et al. 2011). We should underline that, in our study, poor evaluation of fitness was not related to negative symptoms, but rather to problems with the control of emotions and body states (*r* = .466, *p <* .001), low evaluation of appearance (*r =* .39; *p =* .001), and a disordered sense of body identity (*r* = .27; *p <* .034). These results highlight the close relationship between physical activity and becoming lost in body experience and dissatisfaction with one’s appearance, at least in young schizophrenic patients. The difference in our results from those obtained by Vancampfort et al. (2010, 2011) may thus be the effect of the specific nature of the groups (with Vancampfort’s study having older patients) or of the tool used. The patients who took part in our study were generally young, and their daily functioning was not marked by the chronicity of illness linked to reduced physical activity. The final aspect of body image studied was appearance evaluation. Schizophrenic patients did not differ in this aspect of body image from healthy controls. Our results agree with those obtained by Koide et al. (2003), who also did not find such a difference when comparing schizophrenic and control groups.

The next aim of the study was to investigate the relationship between the body self and schizophrenia symptoms. None of the body self aspects were related to the negative symptoms measured by PANSS. This result suggests the independence of BS disorder from negative symptomatology in schizophrenia. Positive symptoms were related to disorders in the sense of body identity, an elevated threshold of sensations, the difficulties with regulation of emotions and physical needs, and a negative attitude to one’s appearance. All of these aspects of BS were related to delusions and conceptual disorganization (*r* values range from *r =* 0.29, *p <* 0.05 to *r* = 0.38, *p <* 0.01) and the elevated threshold alone was also related to excitement (*r =* 0.26, *p* < 0.05). This result is similar to that obtained by Koide et al. (2003), who showed that body image pathology was more strongly related to positive symptoms than negative symptoms. A relationship between florid symptoms and body experience disorders is widely described by clinicians (Sass et al. 2013; Raballo 2013; de Haan and Fuchs 2010), but the description of the co-occurrence of positive symptomatology with a diminished sensitivity to external stimuli has not been underlined by those authors. These results allowed us to hypothesize that active psychosis results in a shifting of engagement from the external world (e.g., sensations) to internal delusional processes caused by the illness. In effect, patients may have a diminished awareness of sensations from the environment. A radical form of this process was described by Kraeplin in the context of the lack of sensitivity to bodily discomfort in schizophrenia, expressed in such behaviors as enduring in uncomfortable positions, burning oneself with a cigar, and indifference to flies settling on the eyelids (Kraepelin 1916). However, it should be underlined that our results need to be treated with caution, as the correlations obtained were rather weak, and were not confirmed by the results of regression analysis, which generally did not indicate positive symptoms as significant predictors for body-self disorders.

Regarding the relationship between BS and demographic variables, we noted that age was related only to disturbances of perception, although the correlations were weak and not supported by the results of regression analysis, which suggests caution in interpreting this result. However it cannot be excluded that younger schizophrenic patients experience more disturbances in the basic function of body self—e.g., in the perception of stimuli due to developmental changes in puberty (Lewine 1981; Harrop and Trower 2001). The relationship between functional and identity aspects of BS with years of education is rather surprising. Taking into account the lack of significant relationship between years of education and the course-of-illness variables, we should search for an explanation which does not rely on possible overlapping of these variables. A possible explanation refers to the relationship between education level and social desirability, leading to potential response distortion (Ones et al. 1996). A higher cognitive ability in better-educated patients may lead to self-enhancement, as a consequence of realizing the negative results of being diagnosed as schizophrenic (e.g., stigmatization).

The results obtained in our study support the observations maid by some researchers that men have more positive body images, than women (Demarest and Allen 2000). In our study, women were more dissatisfied with appearance and fitness, than men. Body dissatisfaction among women is explained as the consequence of internalization of society’s beauty standards, linking being a women with attractiveness and thinness (Brennan et al. 2010). Our results suggests that women suffering from schizophrenia are not free from influences of sociocultural discourse which imposes almost unattainable standards of feminine beauty. Moreover female gender was a risk factor for difficulties with the interpretation and regulation of emotions and physical needs. This result suggests that, as with healthy young women (Sakson-Obada 2009), female schizophrenic patients are more exposed to disturbances in the body experience than men.

Our study generally reveals no associations between BS and the course of schizophrenia. The results suggest that disturbances in the BS are relatively independent of the severity and course of schizophrenia. The above conclusion should be treated with caution, however, since this finding might be caused by the specific nature of the patients participating in the study (young with initial diagnosis). In this context, the significant links observed between the number of hospitalizations for other diseases and disturbances of basic function of BS—like perception (both elevated and lowered thresholds of sensation) and regulation of emotion and physical states—were surprising. Interestingly, those patients did not reported any chronic diseases, which could eventually affect the body experience. Schizophrenic patients who suffer from disturbances in perceiving and regulating body experience (e.g., severe pain, blurred vision, and excessive excitement) may have a tendency to understand them in terms of physical illness. Such misinterpretation can promote a search for medical attention, resulting in a large number of nonpsychiatric hospitalizations. This explanation is consistent with the literature on the hypochondriac and cenesthopathic symptoms considered the core symptoms of schizophrenia (Kato and Ishiguro 1997), together with vague complaints of poor physical conditions (Kobayashi and Kato 2004) and pain-like experience. The prevalence of these body disturbances in the early stages of schizophrenia is high (50 %–70 %; Stanghellini et al. 2012, Kobayashi and Kato 2004) and, according to Bräunig et al. (2000) the symptoms may occur 15 years before the manifestation of schizophrenia. Based on the data from our study, we formulate the hypothesis that long before the diagnosis of schizophrenia is made, disorganization of body experience may be in the foreground in some subjects, and may lead to medical consultations and even to hospitalization for somatic-like symptoms.

Moreover, our study did not find any significant impact of the side effect of drug treatment on BS, which is cohesive with the results of other researchers who investigated the influence of antipsychotic treatment on body image (Priebe and Rohricht 2001, Koide et al. 2003).

## Conclusions

Our study leads to several important conclusions. The results revealed that schizophrenia is a disorder rooted in a lack of “anchoring” of the subject in the body, resulting in confusion in body experience on different levels. The most relevant BS disorder in schizophrenia refers to the functions and sense of body identity, the last of which is a consequence of severe disturbances in perception, interpretation, and ability to cope with sensations, emotions, and body states. Negative emotional attitudes towards the body and its characteristics do not appear to be distinctive of schizophrenia. The exception is acceptance of biological sex, but disturbances in this aspect of body image indicate a rejection of the crucial aspects of identity, so it addresses a different psychological problem than dissatisfaction with appearance or fitness. Our results support the idea that schizophrenia is a disorder better characterized by abnormal experience of the body than by body image disturbances, understood as an evaluation of appearance and other body characteristics. The body image aspects (such as appearance and fitness evaluation), extensively explored in the context of body image psychopathology (bulimia, anorexia, and obesity), do not seem to be a key characteristic of schizophrenia, in which the sense of self is disorganized by delusions and hallucinations. The negative emotional attitude towards the body and its characteristics do not appear to be distinctive for schizophrenia.

Additionally, disorders of stimulus perception and in the ability to coping with emotions and physical needs are related to the number of hospitalizations for other diseases. The clinical implications of the results points to the importance of the detection of early signs of schizophrenia related to distorted body experiences, which may be initially regarded as caused by physical conditions. In further research, the exact reason for nonpsychiatric hospitalization should be taken into account.

Moreover, the body self model seems to be useful in describing different body images and body experience disturbances in psychopathology, as it gathers empirical results and clinical observations into one conceptual framework. We are of the opinion that methods other than the declarative method of body self assessment are of crucial importance in the study of the body-mind issue. Introducing parallel methods of assessment (descriptive and experimental), posing a question of coherence, would seem to be worth considering in further studies.

Our study has certain limitations. First, the sample size was small and the clinical group was specific: patients were young and with their initial diagnosis. It cannot be excluded that the results would be different if the study were conducted in an older, chronically ill group, where negative symptomatology is more pervasive. Second, our study does not give a definite answer to the specificity of BS in schizophrenia. To answer this question, comparison with other clinical groups is required. Third, the study is based on the declarative method, which requires a certain level of insight into experience. A combination of declarative and experimental methods in seeking distinctive traits of BS in schizophrenia should be considered.
